# Effects of Allulose vs Aspartame Consumption on Postprandial Glucagon-Like Peptide-1 Profiles and Metabolic Health: Protocol for a Randomized, Crossover, Double-Blind, Placebo-Controlled Trial

**DOI:** 10.2196/81857

**Published:** 2026-02-19

**Authors:** Selina Busch, Paola G Ferrario, Ann-Kathrin Henk, Ann Katrin Engelbert, Oliver Wittek, Stephanie Seifert, Achim Bub, Carina I Mack, Bettina Hieronimus

**Affiliations:** 1 Department of Physiology and Biochemistry of Nutrition Max Rubner-Institut Karlsruhe Germany; 2 Institute of Sports and Sports Science Karlsruhe Institute of Technology Karlsruhe Germany; 3 Department of Safety and Quality of Fruit and Vegetables Max Rubner-Institut Karlsruhe Germany

**Keywords:** allulose, aspartame, gut hormones, insulin sensitivity, randomized controlled study, satiety

## Abstract

**Background:**

Excessive sugar consumption is a public health concern. Allulose, a low-calorie sugar with similar functional properties to sucrose, offers potential metabolic benefits. Animal and limited human studies suggest it may stimulate glucagon-like peptide-1 (GLP-1) secretion, improve glucose regulation, and support weight management. However, evidence to substantiate these effects in humans remains scarce.

**Objective:**

The primary aim of this study, the low-calorie sweetener intervention study allulose (LisA), was to assess differences in the postprandial GLP-1 profile (primary outcome) between an acute intake of allulose and aspartame interventions in healthy adults. Secondary goals included exploratively assessing potential subacute adaptation effects over a 4-week consumption period and evaluating a comprehensive set of parameters as hypothesis-generating findings for future large-scale research.

**Methods:**

We conducted a randomized, double-blind, placebo-controlled, crossover trial in healthy adults. Participants daily consumed either 3 allulose-sweetened or aspartame-sweetened beverages for 4 weeks in crossover, with a washout in between. Standardized inpatient procedures were conducted at the study baseline and at the beginning and end of each intervention phase. The primary outcome is the postprandial profile of GLP-1. Secondary outcomes include further parameters of gut hormone secretion, insulin sensitivity (Matsuda Index), body composition (body impedance analysis), subjective satiety (visual analog scales), and gastrointestinal tolerance. We also assess multiomic endpoints, including sugaromics and gut microbiome composition. The primary outcome will be analyzed using the incremental area under the curve with a 2-tailed paired *t* test. All further outcomes (including peak and total area under the curve for GLP-1) will be assessed using linear mixed models.

**Results:**

A total of 10 participants (4 female and 6 male; mean age 31.2, SD 6.8 years; BMI 25.1, SD 2.6 kg/m^2^) completed all study procedures. The sample collection phase was successfully concluded in November 2023. Data processing and statistical analysis for the primary outcome are expected to be completed by June 2026.

**Conclusions:**

The comprehensive study protocol, integrating a rigorous crossover design with multiomic analysis, is poised to provide confirmatory evidence for the acute GLP-1 effects of allulose and generate valuable mechanistic hypotheses regarding its subacute metabolic and gut health effects. The findings will contribute to the evidence base required for evaluating allulose’s potential role in public health sugar reduction strategies.

**Trial Registration:**

German Clinical Trials Register DRKS00028521; https://drks.de/search/en/trial/DRKS00028521

**International Registered Report Identifier (IRRID):**

DERR1-10.2196/81857

## Introduction

The dietary intake of free sugars consistently exceeds recommended maximum levels across all age groups in Germany [1-3]. This is a growing public health concern, as excessive sugar consumption—especially from sugar-sweetened beverages—is linked to dental caries, overweight, obesity, and associated metabolic diseases [4,5]. To mitigate these risks, the use of low-calorie sweeteners has increased in recent years, with further growth anticipated [6].

One promising low-calorie sweetener is allulose (D-psicose), a rare naturally occurring sugar found in small quantities in dried fruits and high-sugar processed food [7]. Allulose offers significant calorie reduction, providing only 0.2-0.4 kcal/g, or 5%-10% of the energy content of caloric sugars [8], while maintaining similar functional properties, such as browning, texture, and bulk addition. This makes allulose a promising candidate for sugar reduction strategies. Like its epimer fructose, allulose is absorbed primarily via glucose transporter 5 transporters [9]; however, it is believed to be excreted unmetabolized in the urine [10]. Studies suggest it is well-tolerated at moderate doses, with a gastrointestinal tolerance similar to fructose [11].

Beyond its potential to reduce caloric value in sweetened products, animal studies have highlighted further metabolic benefits of allulose. For example, allulose consumption has been shown to stimulate the release of glucagon-like peptide-1 (GLP-1), a gut hormone that plays a critical role in regulating satiety and postprandial glucose metabolism [12,13], in rodents [14,15]. Increased GLP-1 levels have been associated with improved glucose regulation and reduced food intake in rodent models. In humans, fructose induces GLP-1 release, suggesting that allulose may elicit a similar response [16-18]. However, human data are limited to 1 isolated study showing modest GLP-1 increases after administration of allulose by feeding tube without significant effects on satiety or food intake [19], underscoring the need for further investigation. Furthermore, the metabolic and endocrine effects of sweeteners and fermentable carbohydrates can change over time. Studies investigating dietary fibers and specific nutrients suggest that alterations in the GLP-1 response may be subject to physiological accommodation or adaptation following continuous exposure, driven by changes in the gut microbiota or L-cell sensitivity [20]. Crucially, the currently available human data on allulose focus exclusively on acute, single-dose effects [19]. Previous human trials over several weeks have observed no significant change in GLP-1 release following repeated exposure to sweeteners like aspartame or sucralose [21,22], while preclinical animal studies suggest that chronic sweetener intake can modulate GLP-1 dynamics via changes in enteroendocrine signaling or L-cell function [23]**.** There is a significant gap in knowledge regarding whether the immediate postprandial GLP-1 response is sustained, potentiated, or diminished after a period of regular consumption. Therefore, a subacute intervention period, such as 4 weeks, is essential to adequately capture these potential longitudinal adaptation effects on GLP-1 secretion and other downstream metabolic markers.

Beyond its effects on satiety, allulose may influence glucose metabolism independently of GLP-1. Acute intervention trials in humans have suggested that allulose coingested with glucose or mixed meals can modestly reduce postprandial blood glucose responses, although the effects on insulin levels are inconsistent [24-26]. Proposed mechanisms include inhibition of intestinal α-glucosidase activity and enhanced hepatic glucose usage, potentially mediated by glucokinase activation [27]. Animal studies further support these findings, showing increased hepatic glycogen storage and improved glucose tolerance in response to allulose supplementation [27,28]. However, these effects are not well-characterized in humans, and the potential for allulose to aid in blood glucose regulation warrants further exploration. Allulose could also play a role in body weight regulation. In animal studies, allulose supplementation has been shown to reduce diet-induced weight gain, possibly through enhanced satiety [27-33] and reduced energy intake [32]. Preliminary human trials have also reported weight loss and reductions in body fat with allulose consumption, even in the absence of significant changes in total energy intake or physical activity [34,35]. One proposed mechanism contributing to weight loss is a direct effect of allulose on substrate oxidation within energy metabolism. Studies in both rats [32] and humans [36] have demonstrated that allulose consumption increases postprandial fat oxidation, as measured by indirect calorimetry. These findings suggest that allulose may influence body weight beyond its caloric reduction, potentially via mechanisms related to gut hormone modulation or altered glucose metabolism. However, these effects require confirmation in well-controlled, long-term human studies.

In addition to its metabolic effects, allulose may influence the composition and activity of the gut microbiome. The intestinal absorption capacity for allulose is limited [10]. Following oral intake, a portion of the ingested allulose therefore reaches the lower sections of the intestine, where it becomes available for microbial fermentation. Animal studies have shown that the consumption of allulose leads to changes in the composition of the gut microbiota and short-chain fatty acids [37,38]. The digestion of allulose by bacteria requires specific enzymes encoded by a dedicated gene cluster, which is not ubiquitously present across bacterial species [10,39]. The presence of allulose could therefore offer certain bacteria a selective survival advantage and induce a shift in the microbiota composition [40]. However, whether and to what extent allulose induces changes in the complex human gut microbiome remains unknown.

Despite its promise as a low-calorie sugar alternative, the effects of allulose on human metabolism remain incompletely understood [41]. This study aims to address critical gaps by investigating the impact of allulose consumption on GLP-1 secretion, satiety, glucose regulation, and body weight in humans, as well as potential effects on the microbiome. The findings will contribute to the evidence needed to guide the responsible integration of allulose into the food supply.

## Methods

### Ethical Considerations

The study was conducted in accordance with the Declaration of Helsinki and the Good Clinical Practice guidelines. The trial was prospectively registered in the German Clinical Trials Register under the identifier DRKS00028521 (Universal Trial Number U1111-1291-0885) on May 3, 2023. Ethical approval was obtained in April 2022 from the Ethics Committee of the State Medical Chamber of Baden-Württemberg (approval number F-2022-029). All potential participants were thoroughly informed, both verbally and in writing, about the aims, procedures, potential risks, and benefits of the study, and all participants provided written informed consent prior to their inclusion. To safeguard participant data, all data were pseudonymized immediately upon collection. All identifying personal information is stored securely, separate from the study data, and access to the identity key is restricted to authorized personnel in compliance with applicable data protection regulations. Finally, participants received financial compensation (US $850) for their time commitment.

### Outcomes

The primary endpoint of this randomized crossover trial is the acute postprandial GLP-1 profile comparison between the allulose and aspartame interventions. For the primary analysis, the GLP-1 profile will be quantified using the incremental area under the curve (iAUC), as this metric best reflects the net stimulation effect relevant to appetite modulation and glucose regulation. Peak concentration and total area under the curve (AUC) will also be assessed. No minimal important change for within-participant GLP-1 has been established. The clinical significance of the observed changes will be interpreted in the context of available literature. Secondary outcomes comprise a range of parameters assessed following 4 weeks of daily exposure. A central secondary goal is the investigation of potential subacute adaptation effects on the GLP-1 profile by comparing the acute response with the response at the end of the intervention phase. Additionally, comprehensive parameters such as glucose and insulin homeostasis, insulin sensitivity, satiety (visual analog scales [VAS]), body weight and composition, and gastrointestinal tolerance will be evaluated (secondary outcomes). The study also includes exploratory multiomics analyses, namely sugaromics and gut microbiome composition analysis, to generate mechanistic hypotheses for future, larger-scale studies.

### Study Design and Setting

**Figure 1:**
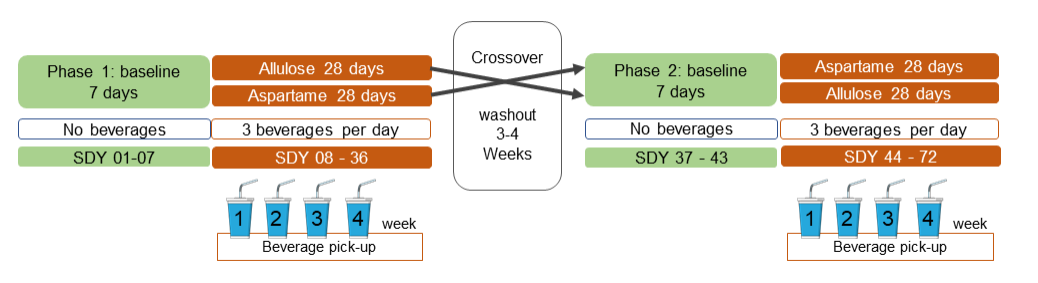
Study schematic: participants were randomly assigned to an intervention sequence. Procedures and study protocol were the same in both intervention phases. SDY: study day.

The study was planned as a randomized, crossover human trial. The study included 2 phases, each consisting of a 7-day baseline period and a 4-week intervention period, separated by a 3- to 4-week washout phase (Figure 1). During each intervention period, participants consumed 3 beverages daily consisting of water sweetened either with allulose or aspartame (control). The sequence of interventions was randomized (allulose–aspartame or aspartame–allulose). Before and during inpatient procedure days (baseline and intervention), participants received a standardized low-sugar dinner. Beverages were consumed with inpatient meals on procedure days, while they were consumed in a private setting on days without procedures. This study protocol was developed in accordance with the SPIRIT (Standard Protocol Items: Recommendations for Interventional Trials) guidelines. The completed SPIRIT checklist is provided as Multimedia Appendix 1 [42].

### Participants

We aimed to include 12 participants (male or female; aged 18‑50 years; BMI 18.5-30 kg/m^2^) with self-reported stable body weight during the prior 6 months. Participants were required to be in good metabolic health, as assessed by the study physician, without nutritional sensitivities (food allergies, fructose malabsorption, celiac disease, irritable bowel disease, etc) and should not show signs of restricted eating (restrain score <13; Fragebogen zum Essverhalten [43], which is the German version of the Three-Factor Eating Questionnaire [44]). Further detailed eligibility criteria are listed in Textbox 1**.**

**Inclusion and exclusion criteria.**

**Inclusion criteria**

- Women and men aged 18-50 years
- BMI 18.5-30 kg/m^2^
- No desired weight change
- Good health
- Written informed consent to participate voluntarily in the study
- No diseases affecting nutrient absorption, digestive function, metabolism, or excretion, in particular malabsorption or intolerance of fructose
- Nonsmokers
- Parameters of liver, kidney, and thyroid function and carbohydrate and lipid metabolism without pathological values (complete blood count, clinical chemical parameters, and preliminary examination)
- No indication of restrictive eating behavior

**Exclusion criteria**

- Pregnant or breastfeeding
- Acute or regular use of medications that affect lipid metabolism, blood glucose regulation, or the gastrointestinal tract
- Use of antibiotics in the past 6 months
- Allergy or intolerance to any food or ingredients in the test meal

Participants were recruited through various channels, including flyers, local and regional newspapers, Instagram, and direct outreach through our internal participant database. Initial screening was conducted through a 20-minute telephone interview, during which participants received an overview of the study and their general eligibility was assessed using a structured interview form.

Individuals meeting the formal inclusion criteria were provided with a copy of the consent form by email, detailing the study objectives and procedures (Multimedia Appendix 2). Interested participants were invited to a screening appointment at the Max Rubner-Institut, where the study principal investigator and study physician explained the study, reviewed the consent form, addressed any questions, and obtained written informed consent from those who chose to participate.

During the screening visit, a fasting blood sample was collected to analyze clinical chemical parameters, blood count, and lipids. Participants also completed the Fragebogen zum Essverhalten [43]. Candidates who met all inclusion criteria and were deemed responsible and likely to adhere to the dietary protocol were invited to participate in the study.

### Intervention

To be labelled “sugar reduced,” a product marketed in the European Union must contain 30% less sugar and energy than comparable traditionally sweetened products [45]. In this study, the amount of allulose was calculated to reflect the substitution of one-third of the sugar in “sugar-reduced” products. Free sugar intake in German adults aged 19-50 years averages 15.3% of daily energy requirements, equivalent to 81.8 g/day [46]. Based on individual daily energy requirements (calculated using the Mifflin equation, with an adjustment of 1.5 for activity), participants received allulose equivalent to 5.1% of their daily energy requirements (15.3% × 1/3), representing a realistic average exposure if one-third of sugar were replaced by allulose upon market introduction. Beverages were prepared on-site by staff who were not otherwise involved in the study.

Aspartame was used in the control group for blinding, as it does not affect the main outcomes assessed in this study [22,47,48]. Beverages were matched for sweetness, and beverage volumes were equal for both interventions. Aspartame concentration was determined by sensory evaluation of sweetness to match the sweetness of the allulose beverage, ensuring effective blinding. Testing was performed by a small group of panelists with prior experience in sensory testing. Allulose concentration was 50 g/L, and aspartame concentration was 113.75 mg/L. Beverage volumes per bottle were adjusted for each participant to match the individual allulose quantity, bottled and frozen at −20 °C until they were handed to the participants.

Participants were asked to consume 3 beverages daily with main meals. Drinks could be flavored to taste (eg, with lemon slices). Participants were instructed to label empty bottles with the consumption date and accompanying meal (breakfast, lunch, or dinner) and return them at the weekly in-person appointments.

The 4-week duration for the intervention was selected to balance participant burden with the physiological need to observe cumulative metabolic and microbial adaptation effects. Furthermore, the washout and intervention periods were chosen to facilitate the testing of female participants during the same phase of their menstrual cycle, thereby reducing a major source of biological variability in the outcomes of interest.

### Randomization and Blinding Procedure

Randomization was performed before study initiation by a staff member who was not involved in participant enrollment or study implementation, ensuring allocation concealment. The 2 intervention sequences—aspartame–allulose and allulose–aspartame—were each entered into stacked columns of 6 cells in a Microsoft Excel spreadsheet. The RAND() function was applied in an adjacent column to generate random numbers, and the RANK() function was used to assign ranks to these numbers. Based on the resulting ranks, the sequences were allocated to participant IDs.

The staff member performing the randomization was also responsible for preparing the drinks and therefore was the only person knowing the sequence allocation during the active phase of the study. Participants were not informed about the sequence of intervention they were receiving. Additionally, both the study staff interacting with the participants and the technical staff analyzing the samples were blinded to the intervention assignments during and after the study.

### Standardized Meals

During the outpatient period, participants were instructed to follow their usual ad libitum diets. On the evenings before study procedures, participants were provided with standardized dinners and were restricted to eating only these meals. The standardized ad libitum meal resembled a typical German cold dinner, consisting of mixed wheat and whole-meal bread, butter, a packet of sliced cheese, cream cheese, a tomato, a cucumber, a carrot, and an apple. Participants were asked to finish eating before 9 PM and to drink only water afterward.

The standardized breakfast consisted of energy-balanced semolina pudding (semolina, milk, cream, and vanilla aroma) providing 25% of the participant’s daily energy requirement. Lunch was a vegetable gratin (pasta, mixed vegetables, cheese, and herb sauce with milk) which was provided ad libitum. These meals contained 50% energy from carbohydrates, 15% energy from protein, and 35% energy from fat. For the inpatient meals, daily energy requirements were calculated by the Mifflin equation [49] with an adjustment of 1.3 for activity, as participants had restricted mobility on the respective days.

### Participant Retention and Follow-Up

Several strategies were implemented to promote participant retention and ensure complete follow-up. Participants received regular personal contact during weekly appointments to return empty bottles and collect the next week’s beverages. This contact provided an opportunity to address any concerns, reinforce adherence, and document any missed doses or adverse effects.

Participants were also reminded of upcoming visits and procedures by email. The study team made every effort to accommodate participants’ schedules and offered flexibility for beverage and meal pickup times.

### Experimental Procedures

#### Anthropometric Measurements and Assessment of Body Composition

Participants were weighed on inpatient days in the morning. Body weight was measured to the nearest 0.05 kg in underwear and was determined on the first and last day of each baseline phase (study day [SDY] 1 and SDY 37; SDY 7 and SDY 44) and at the end of each intervention phase (SDY 35/SDY 71). Height was measured to the nearest 0.1 cm using the Seca 285 measuring station (Seca). Waist and hip circumferences were measured with an ergonomic body circumference measuring tape from Seca. Body composition was measured using bioelectrical impedance analysis (Medical Body Composition Analyzer Seca 515; Seca). Bioelectrical impedance analysis measurement was performed at baseline (SDY 7 and SDY 44) and at the end of each intervention phase (SDY 35 and SDY 71).

#### Satiety Assessment

Satiety was tested and analyzed according to best practice suggestions by experts in the field [50]. Participants were provided with water (baseline) or aspartame- or allulose-sweetened beverages (intervention) as a preload. Fifteen minutes after the preload, participants were provided with a standardized breakfast that had to be eaten within 15 minutes.

Gut hormones will be analyzed in all plasma samples by enzyme-linked immunosorbent assay.

In addition, participants filled out a total of 9 VAS to assess subjective satiety. VAS, 100 mm in length with words anchored at each end, expressing the most positive and the most negative rating, were used to assess hunger, satiation, fullness, and prospective food consumption. The questionnaires were made as small booklets showing 1 question at a time. The first VAS was filled out before the preload and then every 30 minutes until 4 hours after breakfast. Satiety was assessed at 3 timepoints during each study arm: on the first day of the baseline and on the first and last day of receiving intervention beverages in each intervention phase.

Participants were given a standardized lunch on SDY 1 and SDY 37 and SDY 35 and SDY 71 to be eaten in their individual rooms. Participants were served a meal exceeding their calculated lunch energy needs (1.5×), presented in a casserole dish and eaten from dinner plates. They were informed that the portion was too large to be finished. They were asked to fully focus on the meal and not to use any digital device while eating or distract themselves otherwise and continue to eat until they are comfortably satiated. The eating time and amount of food consumed were recorded.

#### 3-Hour Oral Glucose Tolerance Test

Oral glucose tolerance tests (OGTTs) were conducted at baseline (SDY 7 and SDY 43) and on the first day after the 4-week intervention period (SDY 36 and SDY 72). Participants fasted overnight and drank a glucose solution (75 g glucose in 250 mL water) after an initial blood draw. Blood was drawn at 0.5, 1, 1.5, 2, and 3 hours after drinking the glucose solution. Glucose and insulin concentrations will be assessed in all plasma samples. OGTT glucose and insulin AUC and the Matsuda Index for whole-body insulin sensitivity [51] will be calculated.

#### Postprandial Course of Respiration Quotient

Respiratory gas exchange was determined on the first day of the baseline phase (SDY 1 and SDY 37) and on the penultimate day of the intervention phase (SDY 35 and SDY 71) using a calorimetry system (Cosmed Quark RMR; software Omnia 1.6.10). Measurements were conducted before lunch and then once per hour for 3 hours. Measurements were conducted in a quiet room with an initial room temperature of 22 °C. The room was ventilated after each session. Participants wore earplugs to minimize sound exposure. Total energy expenditure, carbohydrate energy expenditure, fat energy expenditure (kJ/min/kg body weight), and the respiratory quotient were calculated.

#### Dietary Intake Assessment (Food Frequency Questionnaires)

Participants filled out food frequency questionnaires (FFQs) at the end of the intervention periods (SDY 35 and SDY 71) after lunch. The FFQ contains 53 food groups and covers the previous 4 weeks and was validated at the Max Rubner-Institut [52]. Given that the FFQ was designed in 2010 and plant-based alternative products have a higher market share today, we added 12 questions in total that asked for the type and amount of plant-based alternatives for milk or meat-based products.

Participants were instructed in advance on the use of the FFQ by trained staff. Mean dietary intake (g) and total energy consumption (kcal) were calculated from the FFQ data. As FFQs are prone to high interindividual differences [53], percent differences between the 2 phases were compared for each participant.

#### Continuous Glucose Monitoring

A continuous glucose monitoring sensor (FreeStyle Libre Pro iQ Continuous Glucose Monitoring System, Abbott Diabetes Care) was placed on the participants’ upper nondominant arm during each baseline week and the last week of each intervention. Participants carried the sensor during 5 outpatient days when food consumption was not controlled and 2 days when they consumed standardized meals. This allowed comparison of glucose responses (24-hour AUC and postprandial responses) under free-living and under controlled conditions, both at baseline and during the intervention. Data were recorded after the OGTT on SDY 8 and SDY 44 and SDY 36 and SDY 72 (FreeStyle Libre Pro iQ Recorder, Abbott Diabetes Care). The participants were blinded to their blood glucose readings. Data were extracted using LibreView Software (Abbott).

### Sample Collection

Table 1 provides an overview of the sample collection timepoints and analyses.

**Table 1:** Sample type and uses.

Sample type and study days		Collection details	Analyses and use
**Blood samples**			
	1, 8, 35, 37, 44, and 71	Satiety assessment: intravenous catheter, samples collected at 0, 0.5, 1, 1.5, 2, 3, 4, and 5 hours	(Gut) hormone analyses (active GLP-1^a^, GIP^b^, CCK^c^, PYY^d^, and insulin), GLUT5^e^ analysis
	7, 36, 43, and 72	OGTT^f^: intravenous catheter, samples collected at 0, 0.5, 1, 1.5, 2, and 3 hours	Glucose, lactate, insulin, metabolomics, and sugaromics
**Urine samples**			
	8, 35, 44, and 71	Spot urine at 0 hours and fractions at 0–2 hours and 2–4 hours	Sugaromics, metabolomics, and microbiome analyses (16S)
	7, 36, 43, and 72	Spot urine at 0 hour and fraction at 0–3 hours	Sugaromics and metabolomics
**Saliva samples**			
	7, 14, 36, 43, 50, and 72	Stimulation using cooled dental wax	Sugaromics and microbiome analyses (16S)
**Stool samples**			
	1–7	Fresh sample processed within 4 hours	In vitro fermentation
	5–7, 41–43, 8–9, 32–36, and 70–72	Frozen sample	Microbiome analyses (16S) and short-chain fatty acids

^a^GLP-1: glucagon-like peptide-1.

^b^GIP: glucose-dependent insulinotropic polypeptide.

^c^CCK: cholecystokinin.

^d^PYY: peptide tyrosine-tyrosine.

^e^GLUT5: glucose transporter 5.

^f^OGTT: oral glucose tolerance test.

#### Blood Samples

##### Satiety Assessment

Satiety assessment was performed on the first day of baseline and on the first and last day of each intervention (SDY 1, SDY 8, SDY 35, SDY 37, SDY 44, and SDY 71). Following insertion of an intravenous catheter, blood samples were collected before the preload (baseline, 0 hour) and then at 0.5, 1, 1.5, 2, 3, and 4 hours. During baseline and on the last day of the interventions (SDY 1, SDY 35, SDY 36, and SDY 71), one additional blood sample was collected after lunch at 5 hours. All blood samples for hormone analysis were collected into chilled K3-ethylenediaminetetraacetic acid blood collection tubes (Sarstedt AG & Co KG) containing 50 µM dipeptidyl peptidase-4 inhibitor (Sigma Aldrich) and 500 KIU/mL aprotinin (Merck KGaA), gently mixed, and stored on ice until centrifugation at 2500 × g for 10 minutes at 20 °C. Serum samples for sugaromics analysis were collected in serum separation tubes (Sarstedt AG & Co KG) at 0, 0 0.5, 1, 1.5, 2, 3, and 4 hours, allowed to clot for 30 minutes, and centrifuged accordingly. All samples were aliquoted and frozen at −70 °C. At 3 hours, additional lithium-heparin plasma samples were collected (Sarstedt AG & Co KG) for glucose transporter 5 analysis. Whole blood was centrifuged at 2000 × g for 10 minutes at 4 °C. Plasma was collected, aliquoted, and stored at −70 °C, and blood cells were processed immediately.

##### Oral Glucose Tolerance Test

OGTTs were conducted on the last day of baseline and 1 day after the intervention (SDY 7, SDY 36, SDY 43, and SDY 72). Following insertion of an intravenous catheter, blood samples were collected at 0, 0.5, 1, 1.5, 2, and 3 hours after glucose ingestion. For glucose (and lactate) analysis, blood samples were collected into GlucoEXACT tubes (Sarstedt AG & Co KG) and centrifuged at 2500 × g for 10 minutes at 20 °C to obtain plasma. Additional serum samples were collected in serum separation tubes (Sarstedt AG & Co KG) in the fasting state and at 0.5 hour and 1.5 hours for metabolome and sugaromics analysis. All samples were aliquoted and frozen at −70 °C.

#### Urine

During satiety assessment (SDY 8, SDY 35, SDY 44, and SDY 71), fasting spot urine samples were collected before ingestion of the allulose drink and as 2-hour fractions after ingestion (0-2 hours and 2-4 hours). Sugaromics will be assessed, and additional metabolome analyses will be conducted if sugaromics results indicate relevant changes.

During OGTT (SDY 7, SDY 36, SDY 43, and SDY 72), fasting spot urine samples were collected before and glucose ingestion, and a 3-hour fraction was collected after glucose ingestion for metabolome and sugaromics analysis. Within 1 hour of collection or end of the collection period, a portion of each sample was centrifuged at 4 °C for 15 minutes at 2500 × g, and 1 mL of supernatant was frozen at −70 °C for subsequent sugar profiling.

For microbiome analysis (16S gene sequencing), participants collected midstream urine after cleaning the area around the ureter opening with a wet cloth on SDY 7, SDY 37, SDY 43, and SDY 71. Samples were put on ice immediately. The remaining urine was incubated at 37 °C for 10 minutes and subsequently centrifuged for 15 minutes at 1500 × g as 2 × 25 or 50 mL depending on the concentration of urine (assessed subjectively according to its color intensity). Supernatants were removed, and the pellets were frozen at −70 °C.

#### Saliva

On SDY 7, SDY 14, SDY 36, SDY 43, SDY 50, and SDY 72, stimulated saliva samples were collected for microbiome and sugaromics analysis. On these SDYs, participants were asked to neither brush their teeth nor use mouthwash nor apply lip balm or similar products in the morning before saliva collection. Before sample collection, participants rinsed their mouth with demineralized water and waited for 10 minutes. During this time participants were not allowed to drink. For collection of stimulated saliva, participants received cooled dental wax (dental oral care orthodontic wax, neutral taste—ARGOMAX) for chewing. Participants chewed the dental wax to stimulate saliva production and collected saliva in tubes cooled on ice for 2 minutes. The collected saliva was weighed to calculate the salivary flow rate. These first samples are used for sugaromics analysis. Afterward, participants received another cooled dental wax and collected 1 mL of stimulated saliva for microbiome analysis while chewing.

#### Stool

##### Sample Collection for In Vitro Fermentation

In vitro fermentation experiments can be used to assess whether bacteria in the samples are capable of metabolizing allulose. Participants were asked to provide 1 fresh stool sample for in vitro fermentation experiments during the course of the first baseline phase (SDY 1-SDY 7). Participants received a prepacked stool collection kit containing a flushable stool sample collection sheet, gloves, a spatula, two 50 mL plastic tubes with perforated caps, an Oxoid AnaeroGen Compact Sachet (Fisher Scientific) to provide an anaerobic atmosphere, and an airtight plastic box. In addition, participants received precise oral instruction and a written manual for the sampling procedure. The stool sample was processed within 4 hours of sampling.

##### Sample Collection for Microbiome Analysis

Fecal microbiota composition will be assessed using 16S ribosomal RNA gene amplicon sequencing, and short-chain fatty acid concentrations quantified by targeted metabolomics. Study participants were asked to provide a total of 10 stool samples for microbiota analyses during the course of the study. Samples were collected during each baseline phase (2 samples within 72 hours each between SDY 5-SDY 7 and SDY 41-SDY 43), during the first days of the intervention (1 sample each between SDY 8-SDY 9 and SDY 44-SDY 45), and at the end of each intervention (2 samples within 72 hours each between SDY 32-SDY 36 and SDY 70-SDY 72). Participants received a prepacked stool collection kit that contained a flushable stool sample collection sheet, gloves, a spatula, 3 small plastic bags, a small plastic container, and 2 cooling packs. In addition, participants received precise oral instruction and a written manual for the sampling procedure. Participants were asked to collect 3 independent, approximately nut-sized aliquots from the same stool, which were placed in a small plastic bag. The small plastic bag was placed in a plastic container and cooled with prefrozen (−20 °C) cooling packs immediately after collection, stored at −20 °C until delivery to the study center, where storage continued at −70 °C.

### Compliance

Participants provided urine samples at weekly visits for beverage pick-up to assess allulose concentrations. As aspartame is efficiently hydrolyzed and absorbed, it cannot be detected in urine samples, therefore lacking the possibility to measure compliance in this intervention directly. Participants were asked to return the empty beverage containers with the time and date of consumption recorded.

### Adverse Events and Safety Monitoring

The participants completed a questionnaire on gastrointestinal symptoms once a week during the intervention phases. The following potential symptoms were recorded: abdominal pain, heartburn, belching, tummy rumbling, bloating, nausea, increased appetite or hunger, decreased appetite or hunger, feeling of fullness, and defecation (diarrhea and constipation). For each of these symptoms, participants were asked whether and to what extent they had experienced it (5-point Likert-scale: none, mild, moderate, intense, and very intense).

In addition to these symptoms, a free text box was provided for any other symptoms to document possible adverse events (AEs). AEs were defined as any health-related occurrences during the intervention phases, regardless of causal relationship to the test substances. All AEs were documented and evaluated by the study physician according to standard criteria (severity, duration, relation to intervention, and outcome). Given the nature of the intervention and the known safety profiles of allulose and aspartame, no serious AEs in connection with their consumption were expected. In case of recurrent or severe AEs, dose adjustment or study withdrawal was predefined.

A blood sample was taken before and after each intervention phase to determine safety parameters. The transaminases gamma-glutamyl transferase and glutamic oxaloacetic transaminase, bilirubin (total), creatinine, uric acid, glucose, hemoglobin A_1c_ (HbA_1c_) and thyrotropin; the electrolytes sodium, calcium, and potassium (all in serum); and a blood count were measured.

### Sample Size Calculation

Sample size was calculated using G*Power 3.1.9.4 with postprandial GLP‑1 profiles as the primary outcome. Based on a paired‑design analysis, our working hypothesis is that allulose produces a higher postprandial GLP‑1 response compared with aspartame. With a significance level of 5% and a power of 90%, a total of 9 participants need to be analyzed. Anticipating a dropout rate of 20%, a total of 12 participants needed to be initially included. The calculation was based on the following assumptions: (1) a comparable effect as seen in Table 1 in Teysseire et al [19] of 1.27 pmol/L, given as Cohen delta after an invention with allulose or water (mean paired difference divided by the SD of the paired differences); (2) a dropout rate of 20%, based on prior studies at the Max Rubner-Institut; and (3) sample size calculation via a 2-tailed paired *t* test due to the crossover design.

### Statistical Analysis

Baseline characteristics of the study population will be summarized using descriptive statistics. Data will be reported as mean (SD) for normally distributed variables and as median (IQR) for ordinal variables.

The primary analysis focuses on the acute difference in the postprandial GLP-1 profile between the allulose and aspartame interventions. For this, the primary outcome, the postprandial GLP-1 profile (as iAUC), will be analyzed using within-subject comparisons inherent to the crossover design. For each participant, paired treatment differences between allulose and aspartame will be calculated from the Δ between the acute postprandial GLP-1 and the corresponding period baseline (Δ[end of intervention − baseline]), adjusted for the corresponding period baseline. The primary treatment comparison will be performed using a paired *t* test. In addition, the magnitude of the within-subject treatment effect will be quantified using Cohen delta. Effect estimates will be presented with 95% CIs. Peak concentration and total AUC for GLP-1, together with the secondary outcomes, including other gut hormones and OGTT parameters, will be treated as exploratory. Postprandial profile data (GLP-1, gut hormones, and OGTT) will be analyzed using linear mixed models. The linear mixed models will include fixed effects for intervention and time (acute vs subacute), with the necessary intervention × time interaction term to assess subacute adaptation effects over the 4-week period. Participant ID will serve as the random intercept, with crossover-sequence as an adjusting variable. Satiety will be assessed as differences from fasting values and analyzed using a comparable mixed-model approach. Dietary intake data from FFQs (eg, kcal or g) may be included as covariates, where appropriate. Further outcomes will be analyzed as absolute or relative changes from baseline (Δ[end of intervention – baseline] or %Δ), using ANCOVA with baseline values and BMI as covariates. All analyses will be conducted using R (R Foundation for Statistical Computing).

No formal correction for multiplicity is planned; the primary outcome will be interpreted as confirmatory, whereas all other outcomes are secondary and will be treated as exploratory. These exploratory analyses will be fully described in subsequent publications focusing on the respective outcomes.

### Data Exclusion

All randomized and compliant participants will be included in the primary and secondary analyses. As there were no major protocol deviations, all participants are considered adherent and will be analyzed accordingly. For continuous outcomes with values below the limit of quantification (LOQ), imputation will be performed using LOQ or LOQ/2. Missing data will otherwise not be imputed, and questionnaire-based outcomes with substantial missingness may be excluded from specific analyses, as needed. Data cleaning procedures will be transparently documented, and any further exclusions will be based on predefined criteria and reported in the respective publications.

### Data Management

All study data will be stored on secure institutional servers maintained at the study site. Access is restricted to study investigators and data managers through individual logins with role-specific permissions. Data are pseudonymized using unique participant codes, and the key linking these codes to personal identifiers is stored securely and separately, accessible only to designated personnel.

Personal information about potential and enrolled participants is collected solely for eligibility assessment and study conduct. Data on screened individuals who did not participate were deleted after documentation of necessary variables for reporting purposes (eg, reasons for exclusion). Throughout the study, all personal information is handled in compliance with applicable data protection regulations to ensure confidentiality before, during, and after trial completion.

For continuous glucose monitoring sensor data, automated digital imports are used to reduce transcription errors. Paper-based records (FFQs, gastrointestinal symptom questionnaires, and VAS) are manually entered using predefined coding schemes. All manually entered data are independently double-checked by a second team member to ensure accuracy and completeness.

## Results

### Trial Implementation

This study was a randomized, crossover human trial conducted at the Study Centre of Human Nutrition, Department of Physiology and Biochemistry of Nutrition, Max Rubner-Institut, Karlsruhe, Germany, from July 4, 2023, to November 16, 2023.

### Recruitment and Retention

**Figure 2:**
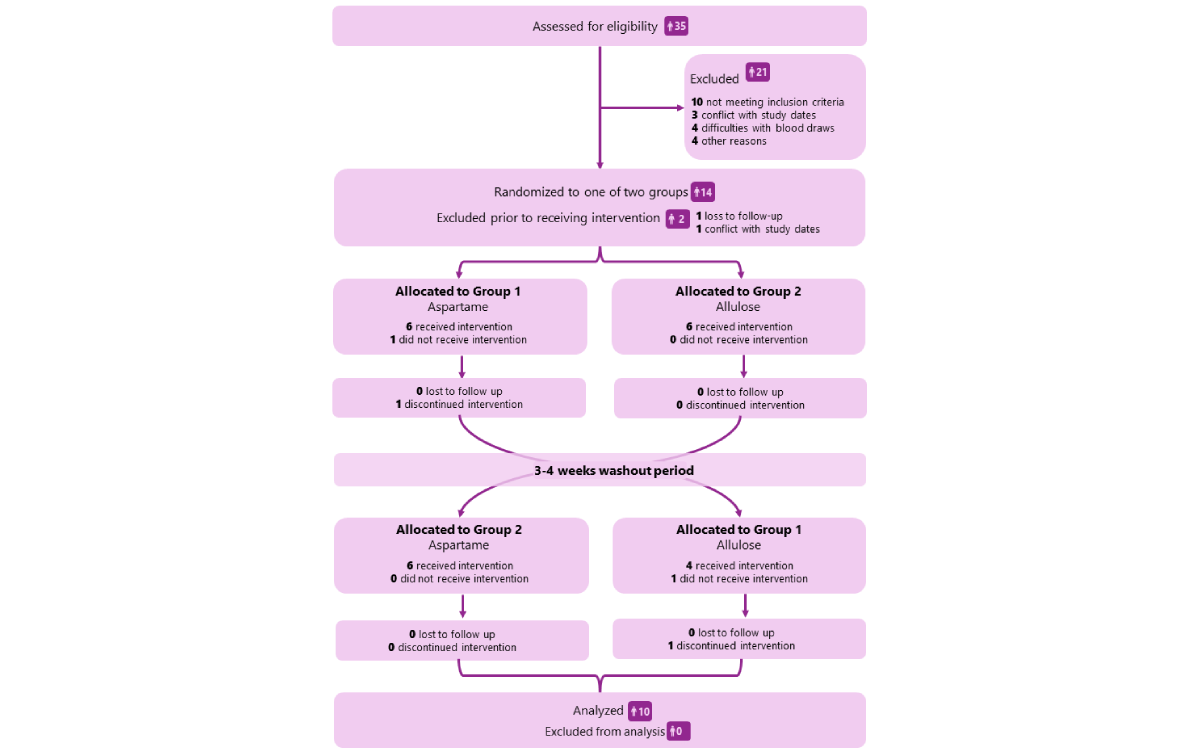
Flowchart of participant screening, exclusions, randomization, and study completion.

An initial telephone interview was conducted with 67 interested individuals, of whom 35 were invited to an in-person screening. We excluded 21 persons, of whom 10 did not meet the inclusion criteria, 4 had difficulties with blood draws (either own discomfort or vein assessment by the study physician), 3 had scheduling issues with our proposed study dates, and 4 had other reasons (did not show up: n=3 and menstrual cycle issues: n=1). Of the 14 invited individuals, 2 dropped out before any study procedures, 1 was lost to follow-up, and 1 person had conflicts with the study dates. Of the 12 participants who started the study, 2 withdrew their participation. One participant discontinued after the first SDY due to discomfort with the intravenous catheters, and 1 participant discontinued after the first SDY of the second study phase due to the time commitment required for study continuation. The unequal loss of participants resulted in 4 participants completing sequence AB (aspartame–allulose) and 6 participants completing sequence BA (allulose–aspartame). No data from these participants were included in the analyses. The participant flow is shown in Figure 2.

### Baseline Characteristics

A total of 10 participants (4 female and 6 male) completed the study. The baseline characteristics of the participants are summarized in Table 2.

**Table 2:** Baseline characteristics (N=10).

Characteristic		Value
Age (year), mean (SD)		31.2 (6.8)
**Sex, n**		
	Female	4
	Male	6
BMI (kg/m^2^), mean (SD)		25.1 (2.6)
Waist circumference (cm), mean (SD)		87.5 (8.1)
Hip circumference (cm), mean (SD)		101.9 (8.0)
Waist to hip ratio, mean (SD)		0.86 (0.1)
Energy requirement (kcal/day), mean (SD)		2481 (295)
Fasting glucose (mmol/L), mean (SD)		4.93 (0.4)
Fasting triglyceride (mmol/L), mean (SD)		1.09 (0.6)
Fasting cholesterol (mmol/L), mean (SD)		4.56 (0.44)
HbA_1c_^a^ (mmol/mol), mean (SD)		32.8 (3.5)

^a^HbA_1c_: hemoglobin A1c.

### Data Status

All biological samples and electronic data records were collected as planned by November 16, 2023. The following key milestones describe the progress of data processing and planned analysis.

Raw data, including all questionnaire responses and physiological readings, underwent a thorough cleaning and curation process, which was finalized in June 2025.

Laboratory analysis for the primary and key secondary outcomes (namely gut hormones and OGTT parameters) were performed in the Laboratory for Molecular Physiology at the Max Rubner-Institut and completed in October 2025. Data cleaning and final curation for these key parameters will be finalized in December 2025. The statistical analysis for the primary outcome and key secondary clinical outcomes is scheduled to commence immediately following final data lock in January 2026.

The exploratory multiomics analyses (sugaromics and microbiome sequencing) are expected to be completed and curated in December 2026, given the complexity and external laboratory processes involved.

We anticipate the main results manuscript, focusing on the primary and key secondary clinical outcomes, to be completed and submitted for publication by June 2026. Analysis and publication of the exploratory multiomics data will follow in separate, subsequent publications.

## Discussion

### Rationale and Significance

This study protocol describes a rigorous, randomized crossover trial designed to evaluate the metabolic and gut health effects of 4 weeks of allulose consumption compared with aspartame. We hypothesize that the consumption of allulose will lead to a measurably higher acute postprandial GLP-1 response compared with the aspartame placebo. We further aim to generate preliminary findings on a potential change in the postprandial GLP-1 response after 4 weeks of consumption and possible effects on insulin sensitivity, body weight and composition, and microbiota composition.

We anticipate that both interventions will be well tolerated, consistent with prior acute studies using similar doses.

This study is poised to provide novel, mechanistic insights by combining continuous glucose monitoring, comprehensive metabolomics (sugaromics), and gut microbiome analysis within a single, controlled, crossover-design pilot. The comprehensive dataset is expected to generate robust parameters for effect size and SDY, which are crucial for designing and powering a definitive, large-scale confirmatory trial on the long-term impact of allulose.

This study addresses the question of whether repeated allulose consumption leads to adaptation of the GLP-1 response, rather than a simple acute effect. Prior human trials have shown no significant changes in GLP-1 after prolonged low-calorie sweetener exposure (aspartame or sucralose) [21]. In contrast, animal models suggest that chronic sweetener intake may dampen the GLP-1 response over time, potentially via alterations in enteroendocrine signaling [23]. By including both acute and 4-week timepoints, our design allows us to explore whether an adaptive incretin response to allulose exists.

The urgency and clinical relevance of these findings have been further underscored by recent regulatory developments. Specifically, the European Food Safety Authority recently indicated that the safety of allulose as a novel food requires additional human data to close identified gaps [54]. Therefore, the results derived from this study will be critical to informing the scientific and regulatory debate surrounding allulose and its potential role in public health sugar reduction strategies.

### Strengths and Limitations

The primary strength of this protocol lies in its robust randomized crossover design, which effectively reduces interindividual variability and increases statistical power. The design, coupled with the final analyzed cohort of 10, is statistically powered to test the primary hypothesis regarding postprandial GLP-1 response, thereby providing a strong foundation for the main outcome. Furthermore, the inclusion of multiomic endpoints (sugaromics and microbiome) alongside clinical measures provides a deep, integrated understanding of the underlying mechanisms.

While sufficiently powered for the primary outcome, the modest overall sample size (N=10) confines the interpretation of all secondary endpoints to hypothesis-generating findings and significantly limits the generalizability of the results. This pilot phase will, however, provide essential data to power future confirmatory studies. Despite the robust design, potential limitations in feasibility could arise, such as participant burden leading to potential nonresponse in questionnaires or technical challenges with complex measurements, such as indirect calorimetry, requiring strict environmental control to ensure reliable data. While double-blind, the differing gut effects of active sweeteners may potentially compromise participant blinding, which could influence subjective outcomes.

### Conclusions

In summary, this protocol describes a methodologically rigorous study designed to investigate the metabolic and gut health effects of allulose consumption. The established methodology and successful data collection are expected to generate critical feasibility and parameter data. The study’s focus on acute postprandial GLP-1 profiles (primary outcome), coupled with comprehensive secondary outcomes such as subacute effects on GLP-1 and other gut hormones, as well as sugaromics and gut microbiome analysis, will provide a novel, integrated understanding of allulose’s mechanisms of action.

## Data Availability

The datasets generated or analyzed during this study are available from the corresponding author on reasonable request.

## Appendix

Multimedia Appendix 1 SPIRIT checklist.

Multimedia Appendix 2 Informed consent form in the original study language (German).

## Abbreviations

AE: adverse event
AUC: area under the curve
FFQ: food frequency questionnaire
GLP-1: glucagon-like peptide 1
HbA1c: hemoglobin A1c
iAUC: incremental area under the curve
LisA: low-calorie sweetener intervention study allulose
LOQ: limit of quantification
OGTT: oral glucose tolerance test
SDY: study day
SPIRIT: Standard Protocol Items: Recommendations for Interventional Trials
VAS: visual analog scales
