# Supplementary material for: Effects of Allulose vs Aspartame Consumption on Postprandial Glucagon-Like Peptide-1 Profiles and Metabolic Health: Protocol for a Randomized, Crossover, Double-Blind, Placebo-Controlled Trial
Source: JMIR Res Protoc. 2026 Feb 19;15:e81857. doi: 10.2196/81857 (PMC12963978; doi:10.2196/81857)
Supplement: Multimedia Appendix 2 [file resprot_v15i1e81857_app2.pdf]

Max Rubner-Institut
Bundesforschungsinstitut für Ernährung und Lebensmittel
Institut für Physiologie und Biochemie der Ernährung
Studienzentrum für Humanernährung
Haid-und-Neu-Str. 9, 76131 Karlsruhe
Telefon: 0721-6625-400, E-Mail: studienzentrum@mri.bund.de

Teilnehmenden-Information

Langzeitinterventionsstudie Allulose (LisA)

Sehr geehrte Damen und Herren,

Sie interessieren sich für die Teilnahme an einer Studie des Max Rubner-Instituts. In dieser Studie soll getestet werden, ob der tägliche Verzehr des seltenen Zuckers Allulose über 4 Wochen einen Einfluss auf Parameter des Stoffwechsels und auf die bakteriellen Gemeinschaften im Körper (Mikrobiota) hat.

Sinn und Zweck dieser Studie

Der Einsatz des seltenen Zuckers Allulose wird als Alternative zu herkömmlichen Zuckern (Haushaltszucker) diskutiert. Allulose kann wie Haushaltszucker verwendet werden, liefert aber nur einen Bruchteil der Energie von herkömmlichem Zucker. Einzelne Studien weisen zudem darauf hin, dass sich der Verzehr von Allulose positiv auf die Sättigung, den Kohlenhydratstoffwechsel und das Körpergewicht im Menschen auswirken könnte. Das wichtigste Ziel dieser Studie ist es deshalb, diese Zusammenhänge zu untersuchen.

Im menschlichen Körper wird Allulose nicht vollständig im Darm aufgenommen und der verbleibende Zucker kann von bestimmten Darmbakterien verwertet werden. Dadurch könnten sich die Verhältnisse der unterschiedlichen Bakterien zueinander verschieben. Daher werden wir auch die Zusammensetzung der bakteriellen Gemeinschaften im Stuhl, Urin und Speichel bestimmen um herauszufinden ob der tägliche Verzehr von Allulose über 4 Wochen einen Einfluss auf die Zusammensetzung hat.

Durch die Teilnahme an dieser Studie erhalten Sie Informationen über Ihr Blutbild und gesundheitsrelevante Körpermaße wie Ihren Body Mass Index (BMI) sowie Taillen- und Hüftumfang. Ferner helfen Sie mit, zu ermitteln wie die Allulose auf den Stoffwechsel wirkt und die Ernährungsforschung im Bereich Zuckeralternativen voranzubringen.

Die Studie wurde der Ethikkommission bei der Landesärztekammer Baden-Württemberg für die berufsrechtliche Beratung der verantwortlichen Studienleitung Prof. Dr. med. Achim Bub (MRI, Institut für Physiologie und Biochemie der Ernährung, Haid-und-Neu-Straße 9, 76131 Karlsruhe) vorgelegt.

Voraussetzungen für die Teilnahme an der Studie

Männer und Frauen im Alter zwischen 18 und 50 Jahren können an der Studie teilnehmen, wenn sie zusätzlich folgende Kriterien erfüllen:

- Body Mass Index (BMI) 18,5 – 30 kg/m^2
- Schriftliche Einwilligung zur Teilnahme an der Studie
- Keine Erkrankungen, welche die Nährstoffaufnahme, die Funktion der Verdauung, die Verstoffwechselung oder die Ausscheidung betreffen, insbesondere keine Unverträglichkeit gegenüber Fruktose
- Die Leber- und Nierenfunktion sowie der Kohlenhydrat- und Fettstoffwechsel dürfen nicht beeinträchtigt sein;
- Keine Diäten zur Gewichtsabnahme
- Nichtraucher und Nichtraucherinnen
- Keine akute/regelmäßige Medikamenteneinnahme mit Auswirkungen auf den Fettstoffwechsel, die Blutzuckerregulation und auf den Magen-Darm-Trakt
- Kein Hinweis auf restriktives Essverhalten
- Keine Einnahme von Antibiotika in den vorrausgegangenen 6 Monaten
- Keine Schwangerschaft oder Stillzeit
- Keine Allergie/Unverträglichkeit gegen Lebensmittel oder gegen Inhaltsstoffe der Studienmahlzeit

Falls Sie in die Studie eingeschlossen wurden, jedoch im Laufe der Studie einer der folgenden Punkte auftritt, können Sie von der Studienteilnahme ausgeschlossen werden:

- Notwendigkeit der Einnahme bestimmter Medikamente im Versuchszeitraum
- Auftreten von Ereignissen, die einer Fortführung der Studienteilnahme entgegenstehen (z.B. Unfall)
- Auftreten von akuten Erkrankungen im Versuchszeitraum
- Widerruf der Zustimmung an der Teilnahme an der Studie
- Nichteinhaltung der Versuchsbedingungen und der erforderlichen Hygienemaßnahmen
- Fehlen relevanter Untersuchungsdaten

Ablauf der Studie

Um festzustellen, ob Sie an der Studie teilnehmen wollen und können, findet ein Termin zur Voruntersuchung statt. Werden Sie in die Studie aufgenommen, erstreckt sich Ihre

Teilnahme über einen Zeitraum von 12-14 Wochen, in denen Sie mehrmals in das Studienzentrum kommen. Damit Sie sich einen Überblick über die Studie verschaffen und den damit verbundenen Zeitaufwand für sich besser einschätzen können, haben wir in einer Übersichtsgrafik auf der letzten Seite dieser Informationsschrift den Ablauf und Umfang der Studie zusammengefasst.

Voruntersuchung: Bei der Voruntersuchung werden wir Ihnen den Inhalt der Studie genau erklären und Sie können Rückfragen stellen. Wir wollen sichergehen, dass Sie genau wissen, was Ihre Studienteilnahme beinhaltet und Sie sich ausreichend informiert fühlen. Sie müssen an diesem Morgen nüchtern in das Studienzentrum kommen und Ihnen werden etwa 12 mL Blut abgenommen. Daraus bestimmen wir Routine-Laborparameter, um sicherzugehen, dass Ihre Blutwerte im Normalbereich liegen. Es wird ein ärztliches Gespräch zu Ihrer Krankenvorgeschichte stattfinden. Diese Voruntersuchung wird an einem Vormittag stattfinden. In der Voruntersuchung überprüfen wir, ob alle Voraussetzungen für Ihre Studienteilnahme erfüllt sind.

Ablauf und Besuche im Studienzentrum: Die Studie umfasst zwei Phasen, die genau gleich ablaufen. In beiden Phasen werden wir zunächst die Ausgangssituation erfassen. In der jeweils ersten Woche bekommen Sie noch keine Allulose oder Kontrollsubstanz, Sie werden aber zu einzelnen Tests zu uns ins Studienzentrum kommen. Im Anschluss erhalten Sie über 4 Wochen Getränke, die Sie dreimal täglich zu den Mahlzeiten trinken. Über diese Zeit werden wir Sie mehrmals ins Studienzentrum bitten, um Getränke abzuholen. Nur zu Beginn und zum Ende der 4 Wochen werden Untersuchungen stattfinden. An die erste Studienphase schließt sich eine 2-4-wöchige Pause an (die sogenannte Auswaschphase) und dann die zweite Studienphase, die genau den gleichen Ablauf wie die erste Phase hat.

Die zwei Studienphasen unterscheiden sich im Testgetränk. Während einer Interventionsphase trinken Sie jeden Tag drei Getränke, die die Testsubstanz (Allulose) enthalten. In der anderen Phase enthält das Testgetränk die Kontrollsubstanz Aspartam, ein kalorienfreier Süßstoff. Die Reihenfolge, in der Sie die unterschiedlichen Getränke erhalten, wird zufällig sein (randomisiert).

Da der Ablauf und alle Messungen in beiden Phasen genau gleich sind, können wir nach Studienende die Effekte der Testsubstanz Allulose auswerten.

Um die oben genannten wissenschaftlichen Fragestellungen beantworten zu können, benötigen wir unterschiedliche Proben und Daten von Ihnen: Diese umfassen mehrere Blutproben, Ihr Körpergewicht und die Körperfettmasse, die Zusammensetzung Ihrer Atemluft und wir werden Sie bitten, Fragebögen auszufüllen. Außerdem benötigen wir Stuhl-, Urin-, und Speichelproben von Ihnen. In diesen Proben sind natürlicherweise Bakterien enthalten und wir werden die Zusammensetzung der bakteriellen Gemeinschaften in diesen Proben bestimmen.

Insgesamt werden Sie über die gesamten 12-14 Wochen 22 Mal an das Studienzentrum kommen. Zwölf dieser Besuche sind nur kurz, hier holen Sie Ihre Testgetränke ab. Für die anderen Termine müssen Sie einen halben (8 Mal) bzw. ganzen Tag (4 Mal) am Studienzentrum einplanen.

Mahlzeiten: Sie werden an mehreren Tagen Mahlzeiten von uns erhalten und Sie werden gebeten, in den entsprechenden Zeiträumen keine weitere Nahrung oder Getränke zu sich zu nehmen. Sie dürfen aber jederzeit so viel Wasser trinken, wie Sie möchten.

Blutglukosesensor: Im Verlauf der Studie wird 4 Mal ein selbstklebender Sensor an einem Ihrer Oberarme angebracht, den Sie jeweils ununterbrochen, also Tag und Nacht, über 8 Tage tragen. Das Anbringen ist schmerzfrei und der Sensor beeinträchtigt Ihren Alltag nicht. Dieser Sensor zeichnet den Verlauf Ihrer Glukosewerte im Gewebe auf und erlaubt uns Rückschlüsse auf Ihre Blutglukosewerte.

Blutabnahmen: An mehreren Studientagen werden wir Sie bitten, morgens nüchtern an das Studienzentrum zu kommen. Über die gesamte Studie werden Sie 10 Mal eine Venenverweilkanüle am Unterarm gelegt bekommen, über die wir zu mehreren Zeitpunkten Blut abnehmen. An diesen Versuchstagen müssen Sie nüchtern kommen, d. h. außer der von uns gereichten Mahlzeit am Vorabend dürfen Sie danach nichts mehr essen und außer Wasser nichts mehr trinken. Bitte trinken Sie am Vorabend auch keinen Alkohol. In den Proben dieses Studientages werden wir Hormone und Stoffwechselprodukte (Metabolite) messen.

Stuhlproben: Wir werden Sie bitten, zu mehreren Zeitpunkten Stuhlproben zu sammeln. Dazu geben wir Ihnen genaue Anweisungen und ein Sammelkit, mit dem Sie bei sich zuhause eine Stuhlprobe sammeln. Diese Probe muss bei Ihnen zuhause eingefroren werden. Sie bringen die Proben dann jeweils beim nächsten Besuch zu uns ans Studienzentrum.

Urin- und Speichelproben: Außerdem werden wir Sie bitten, Urin- und Speichelproben abzugeben. Diese werden am Studienzentrum gesammelt und Sie erhalten genaue Anweisungen, wie Sie jeweils vorgehen müssen. An Tagen, an denen Sie Speichelproben abgeben, dürfen Sie sich vor dem Besuch nicht die Zähne putzen. Die Speichelsammlung findet aber früh am Morgen statt und Sie können sich im Anschluss normal die Zähne putzen.

Atemluft: Wir wollen außerdem nachverfolgen, wie die Nährstoffverwertung in Ihrem Körper verläuft. Dazu nutzen wir die indirekte Messung über eine Atemgas-Analyse. Dazu werden Sie über Mund und Nase eine Maske aufsetzen und in liegender Position für 15min normal atmen. Anschließend erhalten Sie ein Mittagessen und wir wiederholen die Atemgas-Messung einmal pro Stunde, sodass Sie insgesamt 4 Messungen durchlaufen.

Körpermaße und Körperzusammensetzung: Im Rahmen der Studie erfassen wir Ihre Körpergröße und bestimmen Ihr Gewicht. Hierbei nutzen wir eine moderne medizinische Waage, mit der wir gleichzeitig Ihre Körperzusammensetzung ermitteln können. Dies nennt man bioelektrische Impedanzanalyse. Sie ist ein schmerzfreier Vorgang, bei dem der Widerstand in Ihrem Körper über Elektroden erfasst wird, was Rückschlüsse auf die

Körperzusammensetzung (Fettanteil, Muskelmasse, Körperwasser) zulässt. Diese spezifische Messung führen wir nicht an Ihnen durch, wenn Sie Träger bzw. Trägerin eines Herzschrittmachers, implantierten Defibrillators oder einer aktiven Prothese sind.

Fragebögen: Sie werden Fragebögen ausfüllen, die uns Auskunft darüber geben, ob Sie die Getränke gut vertragen und wie hungrig bzw. wie satt Sie sind. Das Ausfüllen dieser Fragebögen wird jeweils nur wenige Minuten dauern. Zusätzlich füllen Sie zu zwei Zeitpunkten zu Hause einen Fragebogen aus, über den wir Ihre Ernährung der vorangegangenen 4 Wochen erfragen. Das wird jeweils ca. 30-45 min in Anspruch nehmen.

Getränke: Über die Interventionsphasen werden Sie Getränke von uns zu trinken bekommen, die Sie zu den Hauptmahlzeiten (Frühstück, Mittag- und Abendessen) komplett trinken sollen. Sie erhalten jeweils genug Getränke für eine Woche, die Ihnen gefroren ausgehändigt werden und wir bitten Sie, diese im Kühlschrank aufzubewahren. Wenn Sie ungern Kaltes trinken, können Sie die Getränke für die jeweilige nächste Mahlzeit ein paar Stunden vorher aus dem Kühlschrank nehmen. Diese Getränke sind in einer Phase mit Allulose gesüßt und in der anderen Phase mit Aspartam.

Blutmengen: Bei der Voruntersuchung werden aus Ihrer Armvene ca. 12 mL Blut entnommen. Über den gesamten Studienzeitraum (inkl. Voruntersuchung) werden Ihnen im Laufe von 12-14 Wochen ca. 432 mL Blut entnommen. Zum Vergleich: Bei einer Blutspende werden an einem einzigen Tag ca. 500 mL Blut abgenommen.

Bitte beachten Sie, dass Sie im Rahmen der Studie eine besondere Verantwortung tragen. Durch Ihre aktive Mitarbeit und das Einhalten der geforderten Studienbedingungen tragen Sie wesentlich zum Erfolg der Studie bei. Wir sind bemüht, alles Erforderliche zu Ihrer Zufriedenheit zu tun und zählen auf Ihre verantwortungsvolle Unterstützung.

Alle Untersuchungen und Analysen Ihrer Blut-, Urin-, Speichel- und Stuhlproben werden im Max Rubner-Institut (Standort Karlsruhe), einem klinischen Routinelabor sowie bei Projektpartnern durchgeführt. Die Ergebnisse Ihrer Untersuchungen können Sie von der Studienärztin Dr. Claudia Dörr, erfahren, wenn Sie dies wünschen.

Stichwort „Freiwilligkeit“

Die Teilnahme an dieser Studie ist freiwillig. **Sie können Ihre Einwilligung jederzeit ohne Angaben von Gründen schriftlich oder mündlich zurückziehen**, ohne dass Ihnen daraus ein Nachteil entsteht.

Stichwort „Aufwandsentschädigung“

Sie erhalten für Ihre Studienteilnahme eine finanzielle Entschädigung.

Stichwort „Risiken“

Grundsätzlich kann jeder Mensch zu jeder Zeit eine Allergie gegenüber bestimmten Substanzen entwickeln, also auch gegenüber Lebensmitteln. Dies können wir natürlich auch in unserer Studie nicht ausschließen.

Beim Tragen des Glucose-Sensors am Oberarm kann es zu einer Hautreaktion kommen, die sich z.B. als Rötung oder durch Juckreiz äußert. Sollten Sie dies bei sich beobachten, melden Sie sich bitte zeitnah im Studienzentrum, damit der Sensor entfernt werden kann.

Bei der Blutentnahme können ein Hämatom (Bluterguss), eine Infektion (Entzündung), Thrombose (Blutgerinnsel) oder Nervenschädigung entstehen.

Durch den Verzehr von Allulose könnten Magen-Darm-Beschwerden wie Krämpfe, Blähungen oder Durchfall auftreten.

Grundsätzlich sind Schäden, die im Rahmen der Studienteilnahme entstehen könnten, über die so genannte *Generalhaftung des Bundes* abgedeckt. Sie gilt auch für das Max Rubner-Institut als selbstständige Bundesoberbehörde im Geschäftsbereich des Bundesministeriums für Ernährung und Landwirtschaft. Sie gilt damit auch für diese Studie. Diese Absicherung würde beispielweise auch für Wegeunfälle gelten, die vom und zum Studienzentrum passieren. Kommt es zu Schadensfällen, werden bei der *Generalhaftung des Bundes* die anfallenden Ausgaben aus dem Bundeshaushalt geleistet. Bitte wenden Sie sich im Schadensfall umgehend an das Studienzentrum für Humanernährung des Max Rubner-Instituts (Telefon: 0721-6625-400; studienzentrum@mri.bund.de).

Stichwort „Datenschutz“ und „Arztgeheimnis“

Die Vorschriften über die ärztliche Schweigepflicht und den Datenschutz werden im Rahmen dieser Studie eingehalten.

Das MRI nimmt den Schutz Ihrer informationellen Selbstbestimmung sehr ernst und hält sich bei der Verarbeitung strikt an die rechtlichen Vorgaben, die über die Datenschutzgrundverordnung (DSGVO) und das Bundesdatenschutzgesetz (BDSG) geregelt sind. Die nachfolgende Erklärung gibt Ihnen einen Überblick darüber, wie wir das konkret in Bezug auf Ihre Studienbeteiligung umsetzen:

Verarbeitung Ihrer Daten

Die Namen aller Studienteilnehmenden und alle anderen vertraulichen Informationen unterliegen der ärztlichen Schweigepflicht.

Rechtsgrundlage für die Datenverarbeitung ist Ihre freiwillige Einwilligung (Art. 6 Abs. 1 Buchst. a, Art. 9 Abs. 2 Buchst. a DSGVO)). Ausdrücklich weisen wir Sie darauf hin, dass Gesundheitsdaten zu der Kategorie besonders schützenswerter personenbezogener Daten gehören. Unbedingt muss gewährleistet sein, dass Ihr Einverständnis zur Teilnahme auf

freiwilliger Basis erfolgt. Dafür ist es erforderlich, dass Sie alle Fragen beantwortet bekommen haben und Sie sich vollumfänglich informiert fühlen.

Der Verantwortliche für die Datenverarbeitung ist der Studienleiter:

Prof. Dr. med. Achim Bub (Achim.Bub@mri.bund.de)

Die Verarbeitung Ihrer personenbezogenen Daten erfolgt zum Zweck der Beantwortung wissenschaftlicher Fragestellungen. Alle im Verlauf der Studie erhobenen Daten werden entsprechend der DSGVO so früh wie möglich *pseudonymisiert* verarbeitet. Forschungsdaten und Proben werden nur in *pseudonymisierter* Form an Projektpartner weitergeben. *Pseudonymisieren* bedeutet, dass die personenbezogenen Daten wie der Name und das Geburtsdatum nicht mehr einer konkreten Person zugeordnet werden können. Die personenbezogenen Daten werden durch einen Nummern- und/oder Buchstabencode ersetzt. Im Studienzentrum des Max Rubner-Instituts in Karlsruhe ist eine Liste hinterlegt, auf der die Namen den Nummern- und/oder Buchstabencodes zugeordnet sind. Diese Liste wird im Studienzentrum gesondert aufbewahrt und unterliegt dort technischen und organisatorischen Maßnahmen, die gewährleisten, dass die personenbezogenen Daten durch unbefugte Personen nicht zugeordnet werden können. Zugriff auf die personenbezogenen Daten haben dadurch nur die zuständigen Personen im Studienzentrum.

Löschung von Daten

Ihre personenbezogenen Daten werden zehn Jahre nach Beendigung oder Abbruch der Studie aufbewahrt und danach gelöscht. Sie sind gegen unbefugten Zugriff gesichert.

Die Codierungsliste, die eine Zuordnung des studienbezogenen Nummern- und/oder Buchstabencodes zu Ihrer Person ermöglicht, wird in jedem Fall zehn Jahre nach Beendigung oder Abbruch der Studie gelöscht, so dass keinerlei Bezug zu Ihrer Person nach diesem Zeitpunkt mehr herstellbar sein wird.

Um Sie zwecks einer Teilnahme an weiteren Studien kontaktieren zu können, archivieren wir Ihre Kontaktdaten (Name und Anschrift) auch über diesen Zeitraum hinaus, jedoch nur, falls Sie uns hierfür Ihre spezifische Einwilligung geben (Einwilligungserklärung „Speicherung von Namen und Anschrift“).

Sind mit der Datenverarbeitung Risiken verbunden?

Bei jeder Erhebung, Speicherung, Nutzung und Übermittlung von Daten bestehen Vertraulichkeitsrisiken (z.B. die Möglichkeit, die betreffende Person zu identifizieren). Diese Risiken lassen sich nicht völlig ausschließen und steigen, je mehr Daten miteinander verknüpft werden können. Der Leiter der Studie versichert Ihnen, alles nach dem Stand der Technik Mögliche zum Schutz Ihrer Privatsphäre zu tun und Daten nur an Stellen weiterzugeben, die ein geeignetes Datenschutzkonzept vorweisen können (z.B. das mit uns kooperierende MVZ

Labor PD Dr. Volkmann und Kollegen in Karlsruhe).
Medizinische Risiken sind mit der Datenverarbeitung nicht verbunden.

Welche Rechte habe ich bezogen auf den Datenschutz?

Sie haben das Recht, Auskunft über die zu Ihrer Person gespeicherten Daten (einschließlich der kostenlosen Überlassung einer Kopie der Daten) zu verlangen. Ebenfalls können Sie die Berichtigung unzutreffender Daten sowie gegebenenfalls eine Übertragung der von Ihnen zur Verfügung gestellten Daten und die Einschränkung ihrer Verarbeitung verlangen.

Ihr Einverständnis zur Verarbeitung Ihrer personenbezogenen Daten können Sie jederzeit widerrufen. Wenn Sie Ihre Einwilligung widerrufen, werden keine weiteren Daten mehr erhoben. Die bis zum Widerruf erfolgte Datenverarbeitung bleibt jedoch rechtmäßig. Sie können im Falle eines Widerrufs auch die Löschung der bis dahin erhobenen Daten verlangen, soweit nicht rechtliche Dokumentationspflichten entgegenstehen.

Ihr Auskunftsrecht, Ihr Recht auf Löschung oder Ihre Widerrufserklärung können Sie an nachfolgende Ansprechpartner richten:

Studienleiter und Studienarzt: Prof. Dr. med. Achim Bub; Achim.Bub@mri.bund.de

Studienzentrum: Datenauskunft.Studienzentrum@mri.bund.de

Max Rubner-Institut
Institut für Physiologie und Biochemie der Ernährung
Studienzentrum für Humanernährung
Haid-und-Neu-Str. 9
76131 Karlsruhe
Telefon: 0721-6625-400

Bei Anliegen zur Datenverarbeitung und zur Einhaltung der datenschutzrechtlichen Anforderungen können Sie sich auch an die **Behördlichen Datenschutzbeauftragten des MRI** wenden:

Telefon: 0721-6625 – 299

E-Mail: datenschutz@mri.bund.de

Sie haben außerdem ein **Beschwerderecht** bei den Aufsichtsbehörden für den Datenschutz:

Bundesbeauftragte für den Datenschutz und die Informationsfreiheit:

Anschrift: Graurheindorfer Str.153, 53117 Bonn

Telefon: 0228-997799-0
E-Mail: poststelle@bfdi.bund.de
Informationen unter: https://www.bfdi.bund.de/DE/Service/Kontakt/kontakt_node.html

Ansprechpartner für Fragen zur Studie

Wenn Sie organisatorische oder inhaltliche Fragen zu dieser Studie haben, wenden Sie sich bitte an:

Dr. Bettina Hieronimus
Telefon: 0721-6625 - 349
E-Mail: bettina.hieronimus@mri.bund.de

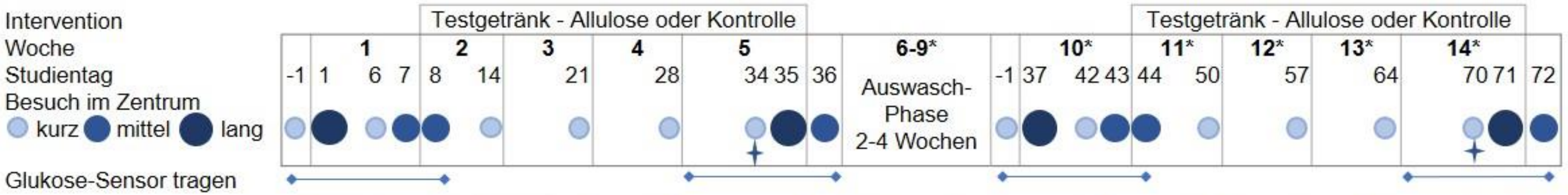

* Maximale Anzahl der Wochen. Kann sich je nach Länge der Auswasch-Phase (mindestens 2, maximal 4 Wochen) unterscheiden.

Kurzer Besuch (ca. 30 min):

- Abholen der Testgetränke und/oder Mahlzeiten
- Abgabe Urinprobe und/oder Speichelprobe
- Mitbringen einer Stuhlprobe
- Bestimmung Körpergewicht oder Körperzusammensetzung
- Fragebogen zu möglichen Symptomen

Mittlere Aufenthaltszeit (ca. 4h Versuchszeit):

- Mehrere Blutabnahmen für Glukosetoleranztest oder Sättigungstest
- Bestimmung Körpergewicht oder Körperzusammensetzung

Vor und an diesen Tagen erhalten Sie Mahlzeiten von uns und Sie dürfen nur diese essen.

Langer Aufenthalt (ca. 8h Versuchszeit):

- Bestimmung Körpergewicht oder Körperzusammensetzung
- Mehrere Blutabnahmen für Sättigungstest
- Messung Ihrer Atemgase

Vor und an diesen Tagen erhalten Sie Mahlzeiten von uns und Sie dürfen nur diese essen.

✦ *an diesem Tag zusätzlich zu Hause:* Ausfüllen eines Fragebogens zu Ihrer Ernährung der vorangegangenen 4 Wochen (Zeitbedarf ca. 30-45 min)

Die angegebenen Untersuchungen finden mindestens an einem der jeweiligen Termine statt. Details können Sie dem Text entnehmen.
